# Yuvezzi™ (carbachol and brimonidine tartrate) dual-agent eyedrops: a new option for presbyopia

**DOI:** 10.1097/MS9.0000000000004984

**Published:** 2026-04-14

**Authors:** Muhammad Ali Abid, Muhammad Hafi Abid, Muhammad Daniyal Afzal

**Affiliations:** a Department of Medicine, King Edward Medical University, Lahore, Pakistan; b Department of Medicine, Faisalabad Medical University, Faisalabad, Pakistan; c Department of Medicine, Kyrgyz State Medical Academy, Bishkek, Kyrgyzstan

**Keywords:** brimonidine tartrate, carbachol, miotic agents, presbyopia, visual acuity

## Abstract

Presbyopia is a progressive, irreversible age-related decline in ocular accommodation caused by reduced lens elasticity, resulting in impaired near vision and a significant impact on daily activities and quality of life. Globally, presbyopia affects approximately 1.8 billion individuals, with projections rising to 2.1 billion by 2030. Current management options include spectacles, contact lenses, pharmacologic agents such as pilocarpine, and surgical interventions; however, each approach carries limitations related to convenience, adaptability, or visual compromise. Yuvezzi™ (carbachol 2.75% and brimonidine tartrate 0.1%) is a recently U.S. Food and Drug Administration (FDA)-approved dual-agent ophthalmic solution designed to improve near vision through pharmacologically induced miosis. Carbachol, a parasympathomimetic agent, stimulates muscarinic receptors to produce sustained pupillary constriction, while brimonidine, an α2-adrenergic agonist, inhibits iris dilator activity and prolongs the miotic effect, enhancing tolerability. Its approval was based on two Phase 3 randomized controlled trials, BRIO-I and BRIO-II, which demonstrated significant improvement in binocular uncorrected near visual acuity without compromising distance vision. Common adverse effects included ocular discomfort, irritation, visual disturbances, and headache. Yuvezzi™ offers a noninvasive, reversible alternative for presbyopia management; however, long-term safety and sustained efficacy require further evaluation.

*Dear Editor,*

Presbyopia is a progressive, irreversible, age-related loss of accommodation of the eye, which results in difficulty focusing on near objects, the cause of which is the decline in elasticity of the ocular lens^[^1^]^. Accommodation declines with age, and by ages 45–55, the remaining accommodation range is about 1.5 diopters (D)^[^1^]^. Presbyopia can significantly impact quality of life, like difficulty reading, double vision, and eye strain^[^2^]^. This also includes tasks that require seeing fine details like threading a needle^[^2^]^. Approximately 1.8 billion people were affected by presbyopia worldwide in 2015, and this number is expected to increase to 2.1 billion by 2030^[^1,2^]^.

The current treatment options for presbyopia include nonsurgical and surgical interventions^[^1^]^. The most common method is optical correction with spectacles or contact lenses^[^1^]^. Pilocarpine, a pharmacological treatment, works by constricting the pupils (miosis) and was approved by the U.S. Food and Drug Administration (FDA) in 2021 for the treatment of presbyopia^[^1^]^. Surgical options include laser-assisted corneal surgery (like laser-induced monovision, multifocal excimer laser ablation, and intracorneal inlays) and lens exchange surgeries (like multifocal lens exchange and extended depth of focus intraocular lens placement)^[^1^]^. All surgical corrections of presbyopia require the patient to accept compromises in flexibility and quality of vision at different distances^[^2^]^.

Recently, the FDA has approved Yuvezzi^TM^ (carbachol and brimonidine tartrate ophthalmic solution) 2.75%/0.1% for the treatment of presbyopia in adults^[^3^]^. Yuvezzi^TM^ is the first FDA-approved dual-agent eye drop designed for presbyopia^[^3^]^. The main mechanism of carbachol and brimonidine is the creation of a pinhole effect through the induction of pupillary constriction, which enhances depth of focus and near visual acuity^[^3^]^. Carbachol, a parasympathomimetic agent, acts as a full agonist at both muscarinic (particularly M3) and nicotinic receptors, leading to strong stimulation of the sphincter muscle and sustained pupil constriction^[^4^]^. This miosis increases depth of focus and improves near visual acuity^[^4^]^. Brimonidine is a selective α2 adrenergic agonist that acts on presynaptic receptors of iris dilator muscles, inhibiting norepinephrine release, hence reducing dilator muscle activity^[^4^]^. Brimonidine not only supports and stabilizes the mitotic effect induced by carbachol but also reduces the side effects of excessive pupil constriction^[^4^]^. Brimonidine helps prolong the duration of the miotic effect induced by carbachol due to its effect on aqueous humor dynamics^[^4^]^.

This approval was given based on results from two Phase 3 randomized, double-blinded controlled trials, BRIO-I (NCT05270863) and BRIO-II (NCT05135286)^[^3,5,6^]^. The study participants were individuals 45–80 years of age, diagnosed with presbyopia^[^3,5,6^]^. BRIO-I (NCT05270863) was a single-dose crossover trial and involved 182 participants^[^3,5^]^. Each participant was given carbachol alone, brimonidine tartrate alone, and Yuvezzi^TM^, with a minimum washout period of 3 days between treatments^[^3,5^]^. BRIO-II (NCT05135286) included 436 participants who were randomly assigned to receive Yuvezzi^TM^ or a vehicle control^[^3,6^]^. The total study duration in BRIO-II (NCT05135286) was 12 months, encompassing a 6-month open-label extension phase^[^6^]^. The primary endpoint in both BRIO-I and BRIO-II was the proportion of patients achieving a gain of ≥3 lines (15 letters) in binocular uncorrected near visual acuity without losing ≥1 line (5 letters) in binocular uncorrected distance visual acuity under mesopic conditions^[^3,5,6^]^.

In BRIO-I, the combination therapy showed greater efficacy compared with brimonidine alone at most timepoints and was significantly better than carbachol alone from 1 to 6 hours^[^3^]^. In BRIO-II, the combination demonstrated a statistically significant improvement in near vision compared to the vehicle at all timepoints^[^3^]^. The most common adverse reactions (incidence ≥5%) are eye pain upon instillation, eye irritation upon instillation, visual impairment, and headache^[^3^]^. The combination is contraindicated in case of hypersensitivity^[^3^]^.

The approval of Yuvezzi^TM^ represents an important addition to the nonsurgical management of presbyopia, providing a pharmacological option that can improve near visual acuity without compromising distant vision. Unlike conventional optical approaches such as glasses or contact lenses, Yuvezzi^TM^ offers a reversible, drop-based therapy that may appeal to patients seeking a noninvasive alternative or those with difficulty adapting to monovision or multifocal lenses. However, the long-term safety, tolerability, and sustained efficacy remain to be fully established, highlighting the need for extended follow-up studies to assess chronic use and real-world outcomes.

## Data Availability

Not applicable to this study.

## Research registration unique identification number (UIN)

Not applicable to this study.
